# Eye Complications in Influenza

**Published:** 1892-02-06

**Authors:** 


### OPHTHALMOLOGY.

### EYE COMPLICATIONS IN INFLUENZA.

The serious ocular complications occur during an attack of influenza has been demonstrated by the large number of cases reported in the medical journals of this, and other countries, and now that we are again visited in this country by an epidemic of the complaint, it is interesting to refer to the various ways in which the eye may be affected by this disease. Principally it is by affecting the central nervous system that the eye becomes involved, just as other nervous complications arise from it, viz., sciatica, chorea, epilepsy, and affections of the pneumogastric, fifth nerve, and the nerves of the bladder, all of which have been reported.

So in the eye affections of the second, third, and sixth nerves arise. The visual troubles are always preceded by violent frontal headache, and gnawing pains around the orbit, but as these pains commonly occur in a case of influenza where visual complications do not arise, they cannot be taken as any special warning of approaching ophthalmic trouble. The usual date of onset of the more serious eye complications is the third or fourth day after the patient has been seized with the pains, one eye only, or both eyes may become affected, and amaurosis may be temporary, or permanent, according to the degree of the nerve affection. The conjunctiva, the cornea, and the deeper parts may all be attacked.

Conjunctivitis is more common as a sequel to an attack than as an accompaniment, and may be confined to the ocular conjunctiva, or include the palpebral also, with inflammation around the roots of the cilia, just as is found following attacks of measles, pertussis, and scarlatina.

Ulcers of the cornea have been found following influenza, as well as an anæsthetic state of the corneal tissues, similar to that produced by cocain. Mr. Higgins has reported two cases of ulcers of the corneas which did not improve with treatment. The one case was also complicated with iritis; there was intense ciliary congestion and a considerable amount of pain. The other was a superficial ulcer, which resisted all treatment from January to April. The patient then went to the seaside till August, when the ulcers were found to be healed, but to have left extensive nebulæ, and even in the following February there was still some opacity.

Case No. 1 had, at the time of reporting, been improved by the use of the actual cautery, but the ulcers had not healed.

Paralysis of accommodation and of all the recti muscles—that is to say, of the third and sixth pair of nerves—have been recorded. This may be unilateral or bilateral, and ptosis and strabismus may be the result.

Several cases of optic neuritis have been met with, in which all other possible causes have been eliminated, such as alcohol, tobacco, and syphilis. These attacks commenced on the third or fourth day after the commencement of the disease, were preceded by violent frontal and pre-orbital pains, and the loss of vision was sudden. The scotoma varied in the cases recorded considerably; neither did it resemble that occasioned by toxæmia from tobacco and alcohol. In some cases only one eye was affected; in the majority both eyes were implicated. In one case recorded the blindness was both complete and permanent. The neuritis appears to be retrobulbar in origin, so that vision may be affected to a serious degree before many signs can be seen in the disc itself with the ophthalmoscope. The patient complains of dimness of vision, and often says he sees a dark cloud before him. When he is tested with Snellen types the vision is found to be much diminished even to V $\frac{5}{0}$ and Jaager's types, J 19 or J 0.

With the ophthalmoscope the optic nerve may be defined, but the edge is softer than usual. There is a slight cloudiness over the vessels near the discs; the veins are large compared with the arteries; and the nerve may appear redder from flushing of the capillaries. These few objective signs, together with much impaired vision and scotomata during or just after an attack of influenza, are sufficient to make it imperative to give a grave prognosis as to the ultimate effect on the sight.

